# Symptomatic Hypoglycemia Related to Inappropriately High IGF-II Serum Levels in a Patient with Desmoplastic Small Round Cell Tumor

**DOI:** 10.1155/2010/684045

**Published:** 2010-06-02

**Authors:** Williams Fernandes Barra, Gilberto Castro, Ana Oliveira Hoff, Sheila A. C. Siqueira, Paulo M. Hoff

**Affiliations:** ^1^Departamento de Oncologia Clínica, Instituto do Câncer do Estado de São Paulo, Faculdade de Medicina da Universidade de São Paulo, São Paulo, Brazil; ^2^Endocrinology, Grupo Fleury, São Paulo, Brazil

## Abstract

A 45-year old man was diagnosed with desmoplastic small round cell tumor (DSRCT) with involvement of the peritoneum and pelvis. Disease progression was observed despite systemic chemotherapy. Six months after diagnosis, he developed severe hypoglycemia presented with seizures. He received intravenous glucose infusion and hydrocortisone with poor glycemic control, but with seizures resolution. The investigation excluded insulinoma, adrenal, liver and GH deficiencies. Laboratory showed slight rise of IGF-II and significant increase of the ratio IGF-II : IGF-I, which is pathognomonic of *non-islet cell tumor hypoglycemia* (NICTH). He received the diagnoses of NICTH related to IGF-II inappropriate production by DSRCT. Despite the attempt to control tumor mass and hypoglycemia, the patient died 9 months after diagnosis. NICTH related to inappropriate IGF-II secretion should be investigated in all cancer patients with refractory hypoglycemia whom insulinoma and other metabolic abnormalities were excluded from.

## 1. Introduction

Symptomatic hypoglycemia can represent a serious complication of malignant neoplasia, usually by inappropriate insulin secretion, which occurs, for instance, in pancreatic islet cell tumor, or unusually by insulin-like growth factors (IGF-I, IGF-II) secreted by tumor cells, referred as *non-islet cell tumor hypoglycemia* (NICTH). Desmoplastic small round cell tumor (DSRCT) is a rare mesenchymal malignant neoplasia with predilection for adolescent and young adults male patients [1]. Here we report a case of an adult male patient diagnosed with DSRCT who developed refractory hypoglycemia.

## 2. Case Report

**Figure 1:**
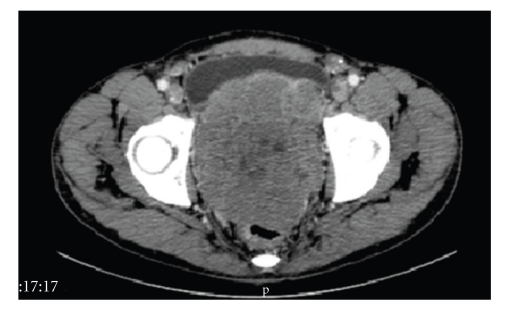
Pelvis CT showing a large pelvic mass.

**Figure 2:**
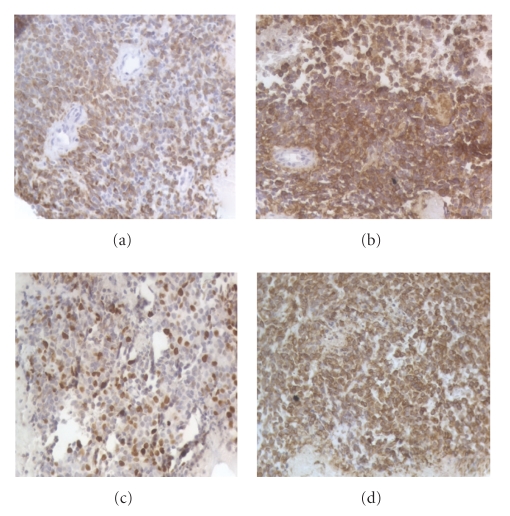
Positive immunohistochemistry for (a) desmin, (b) EMA, (c) Ki-67, (d) vimentin.

**Figure 3:**
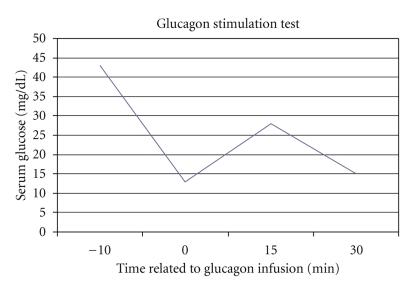
Partial response to glucagon stimulation test.

A 45-year-old man was admitted with a painful pelvic palpable mass and weight loss in November 2006. Pelvic CT scan revealed a 12 × 11 × 12 cm pelvic mass (Figure 1) and several peritoneal lesions. Ultrasound-guided biopsy was performed and cytologic analysis revealed a high-grade mesenchymal neoplasia with small round blue cells. Immunohistochemistry was positive for CD99, enolase, EMA, vimentin, and desmin and negative for 35*β*H11 and AE1/AE3 cytokeratins, chromogranin, synaptophysin, WT1, CD3, CD20, CD30, CD34, S-100, myogenin, and Myo-D1 (Figure 2). These findings were consistent with a pelvic DSRCT with extensive peritoneal involvement. Systemic chemotherapy based on vincristine, doxorubicin, and cyclophosphamide, alternating with ifosfamide and etoposide, was initiated. Disease progression was observed after four cycles. Six months after diagnosis, the patient developed severe hypoglycemia, presenting with seizures. Liver function was normal, and adrenal insufficiency was excluded. He received intravenous glucose infusion and hydrocortisone with poor glycemic control. A glucagon stimulation test, as previously described in [2], was performed with partial response (Figure 3). To better evaluate the etiology of hypoglycemia, the glucose infusion was interrupted for 10 minutes to enable measurements of glucose, cortisol, GH, insulin, C-peptide, IGF-I, and IGF-II (Table 1). With a glucose level of 7 mg/dL, there was appropriate release of cortisol and growth hormone (GH) excluding adrenal and GH deficiency. While C-peptide, insulin levels, and IGF-1 were adequately suppressed, IGF-2 was slightly elevated with increased IGF-II and IGF-I ratio (IGF-II/IGF-I = 22.94) (Table 1) which suggested a paraneoplastic non-islet cell tumor hypoglycemia related to IGF-II overproduction by DSRCT [3]. Despite the attempt to control tumor mass and the hypoglycemia, the patient died nine months after diagnosis.

## 3. Discussion

DSRCT is a rare tumor composed of a desmoplastic stroma with nests of small round blue cells with polyphenotypic differentiation and presenting predominantly mesenchymal differentiation. It has a predilection for adolescent and young adult males and, primarily, involves the abdomen and pelvis [1]. DSRCT is associated with a specific chromosomal translocation, t(11:22) (p13:q12) [4], which juxtaposes EWS gene to the tumor suppressor gene WT1 [1].

It usually presents with advanced disease that must be treated in a multimodal context, based on systemic chemotherapy, radiotherapy, and cytoreductive surgery. The prognosis is usually poor, with few patients achieving long-term survival [1].

The occurrence of tumor hypoglycemia not related to insulin, though rare, has been reported in mesenchymal neoplasms, referred to as *non-islet cell tumor hypoglycemia* (NICTH). Serum levels of insulin and C-peptide must be suppressed to exclude insulinoma [5]. In these patients, an inappropriate (paraneoplastic) tumor production and secretion of insulin-like growth factors have been described. IGF-I and IGF-II interact with peripheral cell receptors (insulin receptor, IGF-I receptor, and IGF-II receptor) leading to hypoglycemia [6]. Both IGF-I and IGF-II circulate almost completely (>90%) bound to specific IGFBPs (IGF-binding proteins). The association of IGFs and IGFBPs with an acid-labile subunit (ALS) results in a ternary complex. This complex has a molecular mass of approximately 150 kDa and is virtually confined to the intravascular compartment [3]. In NICTH, post-translational processing of pro-IGF-II is abnormal [7] resulting in hypersecretion of a higher molecular weight form of IGF-II (“big” IGF-II). “Big” IGF-II is biologically active and present in relatively high amounts in the serum of NICTH patients. In most cases, the serum level of total IGF-II is not elevated indicating that “big” IGF-II must have specific biochemical properties that are different from those of mature IGF-II that lead to an enhanced bioavailability and, consequently, increased insulin-like activity [7]. Treatment should be directed to the underlying neoplasia, with reports of correction of IGF-II levels and normalization of hypoglycemia [8].

In this case, insulinoma was ruled out, and after excluding hepatic, adrenal, and thyroid abnormalities, we detected inappropriately high levels of circulating IGF-II and IGF-II  :  IGF-I ratio, which is virtually pathognomonic of NICTH [3]. We were unable to check for elevation of “big IGF-II” because the assay was unavailable at our center. The glucagon stimulation test is useful to distinguish hypoglycemia mediated by insulin or insulin growth factors from hypoglycemia due to liver failure. In addition, it helps to select patients who may benefit from glucagon treatment. Despite adequate liver function, this patient did not respond appropriately to glucagon stimulation. We believe that the inability to present an appropriate response was probably due to the long refractory hypoglycemia leading to depletion of the hepatic glycogen stores. 

NICTH related to inappropriate IGF-II secretion should be investigated in all cancer patients with refractory hypoglycemia in whom insulinoma and other metabolic abnormalities were excluded.

## Figures and Tables

**Table 1:** Laboratory tests.

Test (unit)	Serum level	Reference
Cortisol (*μ*g/dL)	24	>18 if glucose <40
ACTH (pg/mL)	130	<46
Glucose (mg/dL)	7	70–100
Insulin (*μ*U/mL)	<2.5	To 25
C peptide(ng/mL)	0.6	0.4–3.6
TSH (*μ*U/mL)	3.22	0.4–4.50
T4 free (ng/dL)	1.16	0.7–1.50
GH (ng/mL)	4.2	>3.3 if glucose <40
IGF-I (ng/mL)	65	101–267
**IGF-II (ng/mL) **	**1491**	**414–1248**
**IGF-II/IGF-I ratio **	**22.94**	3.8 ± 1.5
